# Model-based trajectory tracking of a compliant continuum robot

**DOI:** 10.3389/frobt.2024.1358857

**Published:** 2024-04-16

**Authors:** Solomon Pekris, Robert D. Williams, Thibaud Atkins, Ioannis Georgilas, Nicola Bailey

**Affiliations:** ^1^ Department of Mechanical Engineering, University of Bath, Bath, United Kingdom; ^2^ Department of Engineering, King’s College London, London, United Kingdom

**Keywords:** continuum robot, trajectory tracking, robot control, robot modeling, compliant robot

## Abstract

**Introduction:** Compliant mechanisms, especially continuum robots, are becoming integral to advancements in minimally invasive surgery due to their ability to autonomously navigate natural pathways, significantly reducing collision severity. A major challenge lies in developing an effective control strategy to accurately reflect their behavior for enhanced operational precision.

**Methods:** This study examines the trajectory tracking capabilities of a tendon-driven continuum robot at its tip. We introduce a novel feedforward control methodology that leverages a mathematical model based on Cosserat rod theory. To mitigate the computational challenges inherent in such models, we implement an implicit time discretization strategy. This approach simplifies the governing equations into space-domain ordinary differential equations, facilitating real-time computational efficiency. The control strategy is devised to enable the robot tip to follow a dynamically prescribed trajectory in two dimensions.

**Results:** The efficacy of the proposed control method was validated through experimental tests on six different demand trajectories, with a motion capture system employed to assess positional accuracy. The findings indicate that the robot can track trajectories with an accuracy within 9.5%, showcasing consistent repeatability across different runs.

**Discussion:** The results from this study mark a significant step towards establishing an efficient and precise control methodology for compliant continuum robots. The demonstrated accuracy and repeatability of the control approach significantly enhance the potential of these robots in minimally invasive surgical applications, paving the way for further research and development in this field.

## 1 Introduction

Over the 21st century, the inclusion of robotics in the medical field has increased drastically, with far-reaching impact and many novel uses, including fully humanoid robots for homecare (Azeta et al., 2017) and in surgeries (Beasley, 2012). Minimally invasive surgery (MIS), in which a surgeon uses tiny incisions or natural orifices in place of large openings to perform surgery, has particularly benefited from the use of robotics recently due to the precision and control they offer. This approach additionally reduces the length of hospital stay, recovery time, and pain levels throughout compared to traditional surgeries (Fuchs, 2002).

Complaint continuum robots, also known as snake-arm robots or flexible robots, which include end effectors to manipulate and grasp objects, have sparked interest in the surgical field in recent years. They have the potential to reach a surgical site via natural orifices instead of incisions due to their flexible nature, minimizing surgery invasiveness (Burgner-Kahrs et al., 2015), whereas current traditional rigid-link instruments struggle to traverse these pathways while avoiding collisions. Continuum robots for applications in MIS can be actuated through multiple methods, with the most prominent being pneumatic-driven or tendon-driven actuation. Prototype systems have been developed; however, they have yet to reach clinical trials. The majority of research has utilized in vivo or ex vivo tissue experiments or an in vitro environment (Burgner-Kahrs et al., 2015). Nevertheless, these have shown promise in many areas, including colonoscopies (Chen et al., 2009), otolaryngology (Yoon et al., 2011), and cardiac surgery (Gosline et al., 2012).

For a continuum robot to run autonomously, a robust control methodology must be developed. One approach is to utilize a model-based controller, for which a representative mathematical model is required that takes into account the system dynamics. The flexibility of the robot results in it theoretically having infinite degrees of freedom, which leads to modeling complexities that are computationally expensive to solve. Therefore, a compromise between the result accuracy and computation time is usually necessary (Wang et al., 2020). However, for the application of MIS, both accuracy and minimal computation time are necessary. Although some progress has been made, a fully developed solution is not yet available in the open literature.

Multiple approaches to model continuum robots have been studied, with the constant curvature approach and Cosserat theory being the most prevalent. The constant curvature approach is the most common in the literature due to the simplicity of the derived model, resulting in a computational scheme of relatively low complexity (Webster and Jones, 2010). The approach splits the continuum robot into small sections, each of which assumes constant curvature in space. The governing equations are then solved by the first-order method (Burgner-Kahrs et al., 2015). This produces low-accuracy predictions compared to other methods, particularly when torsion, extension, or shear acts on the continuum robot (Sadati et al., 2017). This scenario is likely in surgical application through interactions with tissue or procedures using an end effector. Advancements to improve the accuracy of predictions have been proposed, but they typically involve extremely small segment sizes (Rone and Ben-Tzvi, 2014), and as the constant curvature approach scales poorly in space (Godage et al., 2016), this is not a feasible solution.

Another approach, based on the Cosserat theory, has been growing in popularity due to its high accuracy, making it well-suited for surgical applications. Additionally, it is independent of any discretization strategy, which allows higher-order numerical techniques to be applied, such as a fourth-order Runge–Kutta scheme. Variations of the theory exist, with Rucker and Webster coupling the Cosserat rod and Cosserat string models for a tendon-driven continuum robot, enabling the distributed forces that the tendons apply along the backbone to be accounted for (Rucker and Webster, 2011). A simplified model for the static case of a tendon-driven continuum robot was developed by Isbister et al. (2021), providing a less computationally expensive numerical implementation while maintaining good accuracy. Meanwhile, Renda et al. (2014) presented a dynamic model for a tapered continuum robot based on an octopus arm, showing a good correlation to experimental results. A major drawback of these approaches for the dynamic model is that the derived partial differential equations (PDEs) are computationally expensive to solve due to explicit time integration being implemented, making them difficult to solve in real-time (Alqumsan et al., 2019). Till et al. (2019) proposed a new time discretization scheme where the time derivatives are first semi-discretized outside of a shooting method using implicit differentiation, creating ordinary differential equations (ODEs) in space leading to a boundary value problem. Therefore, any numerical integration scheme utilizing a shooting method or boundary value problem solver can be used at each timestep. The stability of the implicit time discretization allows the robot dynamics to be solved at real-time rates as relatively large timesteps can be used.

A comparison of the representative papers on modeling the forward dynamics of a tendon-driven robot, together with other forms of actuation, is given by Till et al. (2019). However, for use in a feedforward control strategy, the inverse dynamics are needed.

The development of inverse kinematics remains an open problem in the literature, particularly for Cosserat theory. Most studies are conducted using a constant curvature approach, with Jones and Walker using it to form a well-developed formulation of the inverse kinematics; however, it is not in a closed form (Jones and Walker, 2006). On the other hand, Neppalli et al. (2009) developed a closed-form solution for the inverse kinematics, again using the constant curvature approach. However, these still suffer the same inaccuracies as the forward dynamics for the constant curvature approach.

In this paper, the formulation of the dynamic governing equations of a tendon-driven continuum robot is provided in Section 2. An implicit numerical scheme is formulated in Section 3 to provide computationally efficient results. In Section 4, the model is extended to allow for computation of the inverse dynamics, where the input of the model is a demanded continuum robot tip trajectory and the output is the tendon tensions necessary to achieve the demanded motion. This forms the basis of the feedforward control of the tendon-driven continuum robot, which is given in Section 5, together with the details of the experimental facility. The results given in Section 6 show that a 2D trajectory can be accurately tracked to within 9.5% of the movement area for six different demand paths, demonstrating the good trajectory-tracking capabilities of a tendon-driven continuum robot.

## 2 Mathematical model of the continuum robot

**FIGURE 1:**
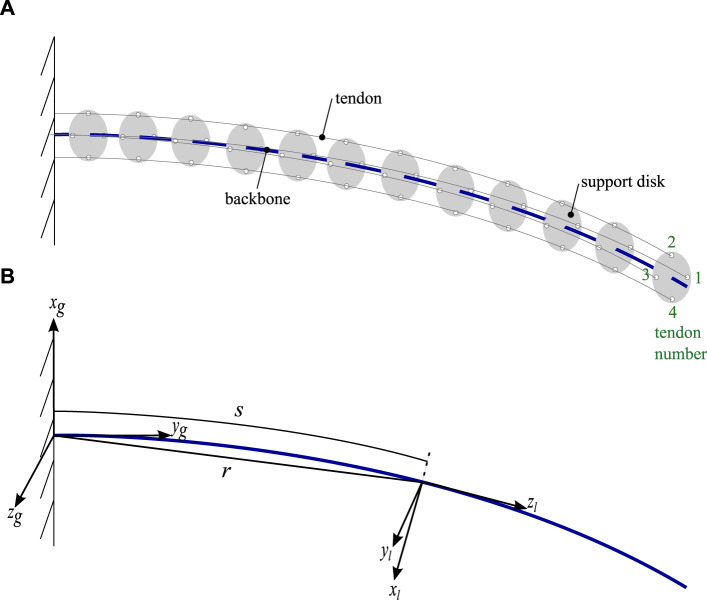
Schematic of the continuum robot showing **(A)** key components including tendon numbering and **(B)** denoting key parameters of the system together with the global and local coordinate systems.

The compliant continuum robot comprises a central backbone with four tendons for actuation, as shown in the schematic in Figure 1A. Two coordinate systems are given: a global coordinate system denoted by subscript *g* and a local coordinate system with subscript *l*. Support disks are used along the backbone to route the tendons parallel to the backbone in the ± *z*_*g*_ and ± *x*_*g*_ directions before being fixed to the last disk. The tendons are numbered counterclockwise when viewed end-on at the continuum robot. The support disks are positioned at equal distances from each other along the backbone.

A dynamic mathematical model for the backbone is derived and coupled to a model for the tendon system, building upon the static model presented by Isbister et al. (2021), following a similar methodology to Rucker and Webster (2011). The governing equations are derived using Cosserat rod theory, where the backbone is considered to be a cantilevered elastic one-dimensional rod, which is assumed to have a constant cross-sectional area that is straight and horizontal when undeformed. The material’s properties are assumed to be perfect. The material damping and drag, together with the inertia and frictional effects from the tendons, are considered negligible. Therefore, all state variables can be parameterized by the reference arc length, *s* and time, *t*. The maximum arc length is *s* = *s*_*m*_, where the length of the backbone is *s*_*m*_ in an undeformed state.

The centroid position along the backbone in global coordinates is given by **r**(*s*) and is shown in Figure 1B together with the arc length, *s*. The local angular and linear rate of change of the backbone position are denoted by **Ω**(*s*) and **u**(*s*), respectively, with the form in Eq. 1.

$$r\left(s\right)=\left(\begin{array}{c} r_{x}\\ r_{y}\\ r_{z} \end{array}\right),\;\;\hat{\Omega }=\left(\begin{array}{c} \Omega_{x}\\ \Omega_{y}\\ \Omega_{z} \end{array}\right),\;\;u\left(s\right)=\left(\begin{array}{c} u_{x}\\ u_{y}\\ u_{z} \end{array}\right).$$ (1)

To transform points from the local to the global coordinate system, the rotation matrix **R**(*s*) is utilized, which is given in Eq. 2.

$$R\left(s\right)=\left(\begin{array}{ccc} \cos\phi \cos\theta & \cos\psi \sin\theta \sin\phi & \cos\psi \sin\theta \cos\phi +\sin\psi \sin\phi \\ \sin\psi \cos\theta & \sin\psi \sin\theta \sin\phi +\cos\psi \cos\phi & \sin\phi \sin\theta \cos\phi -\cos\psi \sin\phi \\ -\sin\theta & \cos\theta \sin\phi & \cos\theta \cos\phi \end{array}\right),$$ (2)

where *ψ*, *θ*, and *ϕ* refer to the angles through which the local axes have turned about the global *x*_*g*_, *y*_*g*_, and *z*_*g*_ axes, respectively.

In the local frame, parameters **v**(*t*) and ***ω*** (*t*), which depend only on time, are defined as the linear and angular velocity of the backbone at position *s*, analogous to **u**(*s*) and **Ω**(*s*), respectively, which have only spatial dependence. Therefore, the derivatives of the position and rotational matrix with respect to the arc length and time are given by

$$\frac{\partial r}{\partial t}=Rv,\;\;\frac{\partial R}{\partial t}=R\hat{\omega },\;\;\frac{\partial r}{\partial s}=Ru,\;\;\frac{\partial R}{\partial s}=R\hat{\Omega },$$ (3)

where $R\hat{\omega }$ denotes the cross product **R** × ***ω***, with the hat representing the skew matrix form in Eq. 4

$$\hat{\omega }=\left[\begin{array}{ccc} 0 & -\omega_{z} & \omega_{y}\\ \omega_{z} & 0 & -\omega_{x}\\ -\omega_{y} & \omega_{x} & 0 \end{array}\right].$$ (4)

Since *∂*(*∂* **r**/*∂t*)/*∂s* = *∂*(*∂* **r**/*∂s*)/*∂t*, using cross-product vector identities, the equations in Eq. 3 can be differentiated to give

$$\frac{\partial v}{\partial s}=\frac{\partial u}{\partial t}-\hat{\Omega }v+\hat{\omega }u,\;\;\frac{\partial \omega }{\partial s}=\frac{\partial \Omega }{\partial t}+\hat{\Omega }\omega .$$ (5)

**FIGURE 2:**
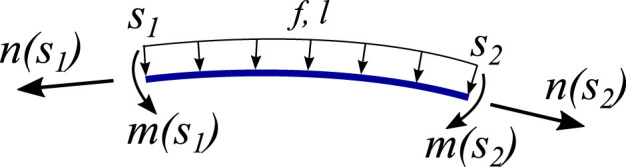
Free-body diagram of an arbitrary section of the backbone between *s*_1_ and *s*_2_ showing the internal forces and moments **n** and **m**, respectively, together with the distributed force and moment **f** and **l**, respectively.

Equilibrium equations can be formed to represent the backbone. In global coordinates, **n**(*s*) and **m**(*s*) are the internal forces and moments within the backbone, while **f**(*s*) and **l**(*s*) are externally distributed force and moment acting on the backbone, respectively, as shown in Figure 2. Therefore, the governing equations are given by

$$\begin{array}{ll} & \frac{\partial n}{\partial s}=\rho AR\left(\hat{\omega }v+\frac{\partial v}{\partial t}\right)-f,\\ & \frac{\partial m}{\partial s}=\frac{\partial }{\partial t}\left(R\rho J\omega \right)-\frac{\partial r}{\partial s}\hat{n}-l=R\rho \left(J\frac{\partial \omega }{\partial t}+\hat{\omega }J\omega \right)-\frac{\partial r}{\partial s}\hat{n}-l, \end{array}$$ (6)

where *A* is the cross-sectional area, *ρ* is the backbone density, and *J* is the matrix of the second moment of inertia tensor. In the case of no actuation forces from the tendons, the only force on the backbone will be from its weight, resulting in **f**_*g*_ = *ρA* **g**, where **g** is the gravitational force. The constitutive law dictates that the internal forces and moments of the backbone can be described using the deformed and undeformed states at arc length *s* along the backbone; the undeformed state is assumed to be a straight cylindrical rod extending in the local *z*-axis defined by **u** ^*^ = (0,0,1)^*T*^ and **Ω** ^*^ = (0,0,0)^*T*^, resulting in Eq. 7

$$n=R\left[K_{se}\left(u-u^{*}\right)\right],\;\;\;\;m=R\left[K_{bt}\left(\Omega -\Omega^{*}\right)\right].$$ (7)

Eq. 8 gives the stiffness matrices *K*_*se*_ and *K*_*bt*_ represent the shear/extension and bending/twisting, respectively, with the form

$$K_{se}=\left[\begin{array}{ccc} G & 0 & 0\\ 0 & G & 0\\ 0 & 0 & E \end{array}\right]A,\;\;\;\;K_{bt}=\left[\begin{array}{ccc} E & 0 & 0\\ 0 & E & 0\\ 0 & 0 & G \end{array}\right]J,$$ (8)

where *E* and *G* are Young’s and shear moduli, respectively.

### 2.1 Coupling the backbone to actuating tendons

The backbone model is coupled to the tendon system, which is used to actuate the continuum robot. A separate reference plane, parallel to the backbone cross-sectional area, is defined for the tendon routing. At any point along the arc length *s*, the offset of a tendon from the backbone in local coordinates is given by $h_{i}\left(s\right)={\left(x_{i}\left(s\right),y_{i}\left(s\right),0\right)}^{T}$ , where *i* = 1, 2, 3, 4 refers to the tendon number, as shown in Figure 1A. In global coordinates, the tendon position is given by **r**_*i*_(*s*) = **R**(*s*)**h**_*i*_(*s*) + **r**(*s*). The tendons are assumed to be perfectly flexible, with negligible shear forces or internal moments. This leads to only tension forces being considered, and as frictional effects are neglected between the tendons and support disks, the tension is constant along the tendon. Both a distributed force/moment along the length of the backbone and point forces/moments, where the tendons attach to the final support disk, are considered.

It is assumed that the distributed force experienced by the backbone is equal to and opposite to the forces generated by the tendons. A static model for the tendons is considered in Eq. 9 with the derivative of the static equilibrium condition for a finite section leading to

$$\frac{\partial n_{i}\left(s\right)}{\partial s}+f_{i}\left(s\right)=0,$$ (9)

where *n*_*i*_(*s*) is the internal force in tendon *i* and **f**_*i*_(*s*) is the distributed force applied. The only internal force in the tendon is considered to be tension, which is denoted by *τ*_*i*_. Eq. 10 shows the internal force being tangential to the backbone at all times, giving

$$\frac{\partial n_{i}\left(s\right)}{\partial s}=\tau_{i}\frac{\frac{\partial r_{i}}{\partial s}}{\left|\frac{\partial r_{i}}{\partial s}\right|}.$$ (10)

Thus, the distributed force **f**_*ten*_ from the tendons on the backbone in the global frame has the form

$$\begin{array}{ll} & f_{\mathrm{ten}}=-\overset{4}{\underset{i=1}{\sum }}f_{i}\;\;\;\;\;\;\;\;\;\;\;\text{where}\;\;\;\;\;\;\;\;\;\;f_{i}=-\frac{\partial n_{i}\left(s\right)}{\partial s}=\tau_{i}\frac{{\frac{\partial \hat{r_{i}}}{\partial s}}^{2}}{|\frac{\partial r_{i}}{\partial s}{|}^{3}}\frac{\partial^{2}r_{i}}{\partial s^{2}}. \end{array}$$ (11)

The total distributed moment experienced by the backbone is the sum of the cross product of each perpendicular distance between the tendon and backbone with each force, resulting in

$$l_{\mathrm{ten}}=-\overset{4}{\underset{i=1}{\sum }}\left(R\left(s\right)h_{i}\times f_{i}\right).$$ (12)

Rewriting the expressions in Eqs 11, 12 in terms of the kinematic variables of the backbone, after considerable algebraic manipulation, gives

$$\begin{array}{l} f_{\mathrm{ten}}=R\left(a+A\frac{\partial u}{\partial s}+G\frac{\partial \Omega }{\partial s}\right),\\ l_{\mathrm{ten}}=R\left(b+B\frac{\partial u}{\partial s}+H\frac{\partial \Omega }{\partial s}\right), \end{array}$$ (13)

where

$$\begin{array}{ll} & a=\overset{k}{\underset{i=1}{\sum }}a_{i},\;\;\;b=\overset{k}{\underset{i=1}{\sum }}b_{i},\;\;\;A=\overset{k}{\underset{i=1}{\sum }}A_{i},\;\;\;B=\overset{k}{\underset{i=1}{\sum }}{\hat{h}}_{i}A_{i},\\ & G=\overset{k}{\underset{i=1}{\sum }}A_{i}{\hat{h}}_{i},\;\;\;H=\overset{k}{\underset{i=1}{\sum }}{\hat{h}}_{i}A_{i}{\hat{h}}_{i},\\ & a_{i}=-A_{i}\left(\hat{\Omega }\left({\left(\frac{\partial r}{\partial s}\right)}^{\zeta }+\frac{\partial h_{i}}{\partial s}\right)+\frac{\partial^{2}h_{i}}{\partial s^{2}}\right),\;\;\;b_{i}={\hat{r}}_{i}a_{i},\\ & {\left(\frac{\partial r_{i}}{\partial s}\right)}^{\zeta }=\hat{\Omega }h_{i}+\frac{\partial h_{i}}{\partial s}+u,\;\;\;A_{i}=-\tau_{i}\frac{{\left({\left(\frac{\partial {\hat{r}}_{i}}{\partial s}\right)}^{\zeta }\right)}^{2}}{{\left|{\left(\frac{\partial r_{i}}{\partial s}\right)}^{\zeta }\right|}^{3}}. \end{array}$$

The superscript *ζ* represents the local frame representation of a variable. Using Eq. 5 and Substituting the equations from Eq. 13 into *∂* **n**/*∂s* and *∂* **m**/*∂s* in Eq. 6 gives the final set of PDEs to describe a tendon-driven continuum robot as

$$\begin{array}{ll} & \frac{\partial r}{\partial s}=Ru,\;\;\frac{\partial R}{\partial s}=R\hat{\Omega },\\ & \left[\begin{array}{c} \frac{\partial u}{\partial s}\\ \frac{\partial \Omega }{\partial s} \end{array}\right]={\left[\begin{array}{cc} K_{se}+A & G\\ B & K_{bt}+H \end{array}\right]}^{-1}\left[\begin{array}{c} c\\ d \end{array}\right]\\ & \frac{\partial v}{\partial s}=\frac{\partial u}{\partial t}-\hat{\Omega }v+\hat{\omega }u,\;\;\frac{\partial \omega }{\partial s}=\frac{\partial \Omega }{\partial t}-\hat{\Omega }\omega , \end{array}$$ (14)

where

$$\begin{array}{ll} & c=\rho A\hat{\omega }v+\rho A\frac{\partial v}{\partial t}-\hat{\Omega }K_{se}\left(u-u^{*}\right)-R^{T}\rho AG-a,\\ & d=\rho J\frac{\partial \omega }{\partial t}+\hat{\omega }\rho J\omega -\hat{\Omega }K_{bt}\left(\Omega -\Omega^{*}\right)-\hat{u}K_{se}\left(u-u^{*}\right)-b. \end{array}$$ (15)

In addition to the distributed forces along the tendon, the point forces are considered at the last support disk where the tendon terminates. A set of forces and moments is applied to the backbone that are equal to and opposite to those internal to the tendon, which have the form

$$\begin{array}{ll} & F_{i}\left(s_{m}\right)=-n_{i}\left(s_{m}\right)=-\tau_{i}\frac{\frac{\partial r_{i}}{\partial s}\left(s_{m}\right)}{\left|\frac{\partial r_{i}}{\partial s}\left(s_{m}\right)\right|},\\ & L_{i}\left(s_{m}\right)=-m_{i}\left(s_{m}\right)=-\tau_{i}R\left(s_{m}\right){\hat{h}}_{i}\left(s_{m}\right)\times \frac{\frac{\partial r_{i}}{\partial s_{i}}\left(s_{m}\right)}{\left|\frac{\partial r_{i}}{\partial s}\left(s_{m}\right)\right|}. \end{array}$$ (16)

These will be applied as a boundary condition when solving the system of equations in Eq. 14. However, the axial point force from the tendons on the last support disk is assumed to be negligible, as it is expected to be at least an order of magnitude smaller (Gravagne et al., 2003).

## 3 Numerical technique

Initially, the PDEs in Eqs 14, 16 are discretized in time using an implicit differentiation formula, resulting in a system of ODEs in space. For a first-order derivative, Eq. 17 shows the general form of grouping of terms dependent on the previous timestep

$$\frac{\partial \gamma^{j}}{\partial t}≃\alpha_{0}\gamma^{j}+\overset{\infty }{\underset{\kappa =1}{\sum }}\left(\alpha_{\kappa }\gamma^{j-\kappa }+\beta_{\kappa }\frac{\partial \gamma^{j-\kappa }}{\partial t}\right)=\alpha_{0}\gamma^{j}+\gamma^{p},$$ (17)

where *γ* ^*p*^ represents the sum of all the terms dependent on previous timesteps and *γ* ^*j*^ represents the sum of all the terms dependent on the current timestep. This approach is applied to the set of PDEs in Eq. 14 to transform it into a set of ODEs. This has the advantage of the time discretization being decoupled from the spatial solver, such that the spatial solver has a new constant value for *γ* ^*j*^ at each timestep, reducing the computations needed at each timestep.

Therefore, the two time derivatives in Eq. 14 become

$$\frac{\partial u}{\partial t}=\alpha_{u}u+u^{p},\;\;\;\frac{\partial \Omega }{\partial t}=\alpha_{\Omega }\Omega +\Omega^{p},$$ (18)

and the time derivative terms *∂* **v**/*∂t* and *∂* ***ω***/*∂t* contained within *c* and *d* in Eq. 15 have a similar form. As a compromise between solution accuracy and computational time, a backward Euler method for the implicit discretization schemes is implemented; numerical damping is considered negligible due to the slow-moving nature of a continuum robot.

Therefore, the system of equations in Eq. 14 has been transformed, through discretization, into a set of ODEs at each timestep. A numerical technique utilizing a shooting method solver based on the fourth-order Runge–Kutta method is implemented. Thus, the boundary value problem is reduced to an initial value problem, where the solution iterates until the boundary conditions are satisfied. A trust-region dog-leg algorithm is utilized to identify if the solution has converged to the prescribed tolerance.

## 4 Trajectory tracking

Currently, the mathematical model has an input of tendon tensions over time, and the output is the backbone position in space and time. However, for a scenario where the end of the backbone is required to follow a given trajectory, the reverse is needed. Therefore, the model is modified to allow a demand trajectory of the backbone tip to be the model input, with the necessary tendon tensions to achieve this demanded trajectory being the model output.

The trajectory demand is given in the *z*_*g*_ and *x*_*g*_ coordinates over a fixed time period, with motion in the *y*_*g*_ coordinate unconstrained. Thus, the demand is given by (*z*_*d*_(*t*), *x*_*d*_(*t*))).

Two additional equations are added to the set of equations in Eq. 14.

$$\frac{dz_{g}}{ds}=0,\;\;\;\;\;\frac{dx_{g}}{ds}=0,$$ (19)

with the corresponding boundary conditions

$$z_{g}\left(s_{m}\right)=z_{d},\;\;\;\;x_{g}\left(s_{m}\right)=x_{d}.$$ (20)

The solution of this extended set of governing equations, Eq. 14, Eqs 18−20, with an initial guess for the tendon tensions at each timestep, will result in the identification of the tendon tensions needed to achieve the demanded trajectory.

## 5 Experimental facility

**FIGURE 3:**
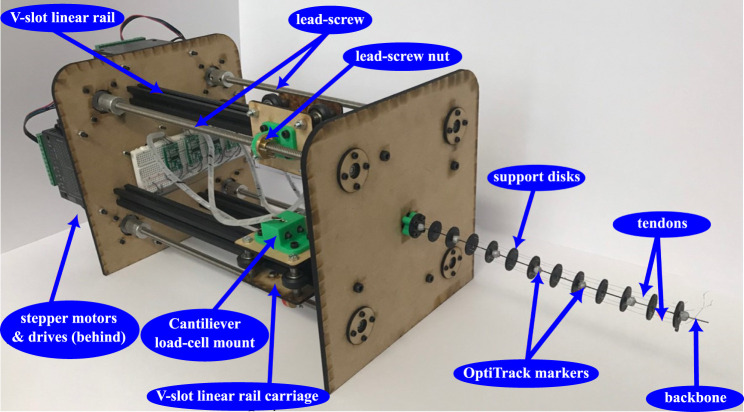
Continuum robot rig where the central backbone is actuated by tendons running through the support disks, driven by stepper motors and lead screws. Cantilever load cells measure the tension in the tendons. OptiTrack spherical markers are positioned along the length of the backbone for position identification.

The tendon-driven continuum robot utilized for the experiments is shown in Figure 3 with details of its manufacturing given by Isbister et al. (2021). Here, a short description of the key elements that are related to the work at hand will be given. The backbone is made from ASTM A228 spring steel, a flexible steel that can bend elastically at high curvatures with a length of *s*_*m*_ = 227 mm. Four 0.28-mm-diameter Dyneema wire tendons, which provide high fracture stress while minimizing possible elongation, run through 12 Teflon-coated acrylic support disks. The support disks are spaced 20 mm apart along the backbone and have a 20 mm diameter and 1.5 mm thickness. The offset from the backbone to the tendon is 8 mm. The Young’s modulus of the backbone was identified to be *E* = 168 GPa, which was found experimentally [Isbister et al. (2021)].

The four tendons are numbered as in the model (Figure 1A) and are actuated independently using lead screw mechanisms driven by stepper motors. Each stepper motor has a resolution of 0.05625°, and combined with the lead screw providing 0.008 m of translation per rotation, this results in a linear resolution of 1.25 × 10^−5^ mm per step. A V-slot linear rail and carriage system is utilized to ensure stability and pure linear translation of the lead screw nut. A benefit of this approach is that minimal friction will occur in the actuation system, which is necessary for the high tendon tension case. A cantilever load cell is fixed to the V-slot linear rail carriage, which is then connected to the tendon, which enables feedback for each tendon. A 1 kg load cell was chosen as a compromise between the maximum expected tendon tension and resolution.

To collect positional data for the backbone using contactless technology, an OptiTrack motion capture vision system was used. A six-camera setup was calibrated to ensure that the maximum tracking error was not more than 0.1 mm. Seven reflective markers of 6 mm diameter were placed along the robot backbone at each tendon support disk for the cameras to track and then compared with the simulation data.

**FIGURE 4:**
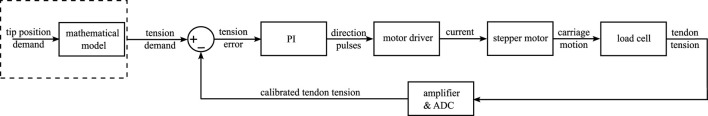
Block diagram of the closed-loop control for regulating the tendon tension. The dashed box gives the additional part of the control scheme needed when the input is the backbone tip position instead of the tendon tension demand. The control loop runs at 100 Hz and is a PI controller based on the error between the demand and tendon tension feedback. The control signals are pulses for the motor driver, which generates the current to run the stepper motor. The lead screw mechanism applies the tension to the tendon via the cantilever load cell. The measurements from the latter are amplified and sampled from the ADC at the rate of the control loop.

The model was run in MATLAB off-line, and the resulting tendon demands for the entire experiment run were sent to an Arduino board. The tendon closed-loop controller was implemented on an Arduino board and ran at 100 Hz. The block diagram of the control system is presented in Figure 4 for both the forward and reverse models. The control variable is the tension of the tendons, *T*_*i*_, with feedback control implemented via a PI controller. The demand values are held via a zero-order hold, and the tension feedback is provided by the load cell via an integrated amplifier/ADC NAU7802, which is sampled at the control loop frequency. The PI gains for the proportional and integral terms were tuned primarily using the heuristic Ziegler–Nichols method (Ziegler and Nichols, 1942) (*K*_*p*_ = 2000 and *T*_*u*_ = 120) to *K*_*p*_ = 2000 and *K*_*i*_ = 20, respectively. If a demand is given for the tendon tensions, the control loop to the right of the dashed box in Figure 4 is implemented. However, for the case of tracking a demanded backbone tip position, the complete control loop in Figure 4 is implemented, with the dashed box generating the required tendon tension from the demanded backbone tip position at each timestep, which is then passed to the low-level controller for tendon control.

## 6 Results

This section describes the experimental results of testing the tracking ability of the continuum robot with the control path derived from the mathematical model.

Six displacement paths were generated, demanding a given position in (*z*_*g*_, *x*_*g*_), i.e., the two global directions perpendicular to the direction along the backbone at its base, as seen in the reference frame in Figure 1A. The *y*_*g*_ direction is left unconstrained and calculated by the mathematical model or observed by the motion of the mechanism in the experiments. In this section, the subscript *g* is omitted as all demands and results are given in the global reference system. The six demands are executed in the top-left quadrant of the robot (as seen from the front) to require the tension of only two of the four tendons, namely, tendons 2 and 3. Three repetitions of each path demand are executed, where the paths are given by

• Horizontal line of length 0.08 m over 20 s.
• Vertical line of length 0.08 m over 20 s.
• Diagonal line of length 0.113 m (covering a vertical and horizontal distance of 0.08 m) over 20 s.
• Circle of diameter 0.08 m over 20 s.
• Square with sides of length 0.08 m over 40 s.
• S-shaped path moving a distance of 0.08 m horizontally and 0.08 m vertically in two, 0.04 m, stages over 100 s.

**FIGURE 5:**
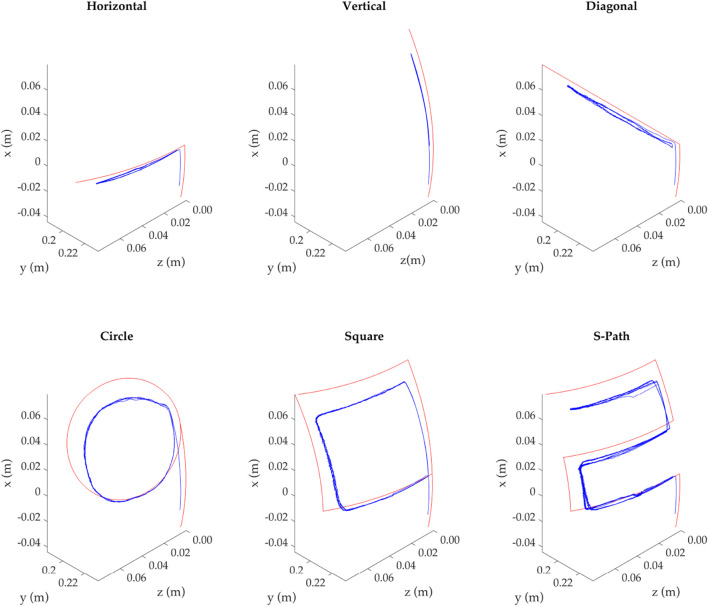
Demand and realized trajectory at the tip of the continuum robot for all investigated paths. All paths are in the same quadrant, upper-left, as seen from the front of the robot, which is plotted here.

Figure 5 presents the tip positional tracking of the simulation, in red, and one of the repetitions of the experimental result, in blue. It should be noted that the simulation result is plotted rather than the demanded path, as both have the same (*z*, *x*) coordinates; however, the *y* coordinate must be found by solving the mathematical model. Thus, the simulation path is the tip-most positional information of the backbone, while the experimental data consists of the tip-most reflective marker 3D information as detected by the optical tracking system (OptiTrack).

The results show that the continuum robot tracks the demanded path; however, the further away from its resting position it becomes, the larger the divergence with the simulation. A potential explanation of this behavior is due to the increased friction caused by the higher tension applied to the backbone, the support disks, and the actuation elements.

**FIGURE 6:**
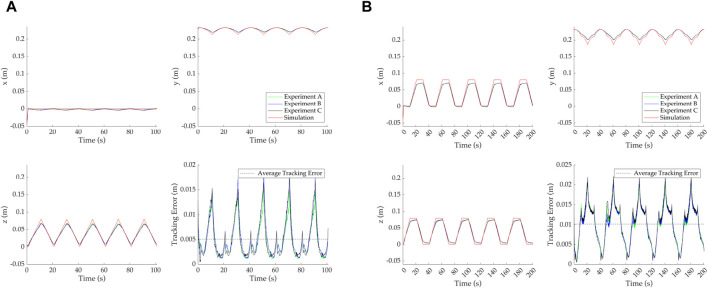
Detailed view of the demand paths and tracking error for *Horizontal* with the minimum average error, shown in **(A),** and the *Square* with the maximum average error, shown in **(B)**. **(A)** Horizontal demand path. **(B)** Square demand path.

Figure 6 shows a detailed view of the demand and each of the three experimental response values for (*z*, *x*) coordinates, as well as the *y* coordinate given by the simulation prediction and experimental results. This is due to *z* and *x* being actively tracked, while *y* is passively affected by the geometry and robot motion. For reasons of clarity, we include only the *Horizontal* and *Square* paths. The tracking error is also presented, calculated as the Cartesian distance between the tip-most OptiTrack marker and the tip-most backbone point from the simulation at each timestep. The average error for all three experiments on a given path is included (as a dashed line) as an easier measure to evaluate performance.

Similar to the overall behavior seen in Figure 5, the tracking error is variable in time and increases as the motion of the continuum robot moves further from the resting position. For example, in Figure 6A, *z* tracking fails to achieve the full desired value at a maximum displacement of 0.08 mm. Similar behavior can be seen for *x* and *z* coordinates in Figure 6B. Moreover, Figure 6A shows that the *x* coordinate also fluctuates for a static demand of 0 N, which implies that the effects of the physical tension application, including calibration and friction, influence the system’s behavior and affect the tracking error.For completeness, the average error for each of the six paths over the three repeats is given in Table 1. Additionally, the error as a percentage of the top-left quadrant diagonal, i.e., 0.113 m, is also given. This metric was selected to allow easier comparison of tracking performance between different demand paths.

**TABLE 1:** Average error for the six demand paths.

Demand path	Average error (10^−3^ m)	Percentage of quadrant diagonal (%)
*Vertical*	7.466	6.61
*Horizontal*	5.048	**4.47**
*Diagonal*	7.974	7.06
*Circle*	8.280	7.33
*Square*	10.47	**9.27**
*S-shape*	8.768	7.76

It can be seen that the minimum average error calculated is achieved by the *Horizontal* path with 5.048 × 10^−3^ m, while the next best performance is the *Vertical* path with 7.466 × 10^−3^ m. Given that both are linear trajectories of 8 mm, the difference can be attributed to the effect of gravity, since the latter path is against it. The trend remains with increasing average error for increasing complexity of motion, with the order being *Diagonal*, *Circle*, *S-shape*, and *Square*. It is observed that the square exhibits the highest average error at 10.470 × 10^−3^ m, and potential reasoning has to do with the fact that the cornering required to achieve the path is the highest of all demands. Nevertheless, the error is still only 9.27% of the quadrant diagonal, i.e., the maximum displacement that can be requested. This value is close to previously reported results (10.46%) for similar mechanism architectures (tendon-driven continuum robot) as compared to a model of a similar modeling approach (forward dynamic Cosserat rod theory model) with similar trajectories (time-varying tension demands for bending) (Renda et al., 2014).

**FIGURE 7:**
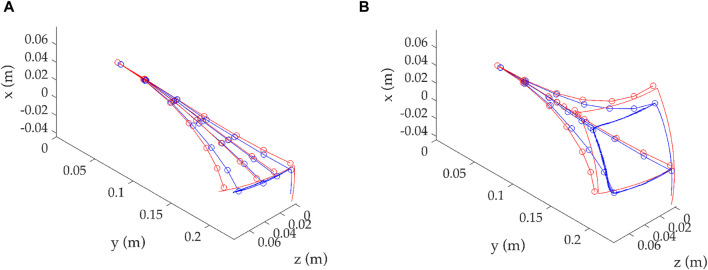
Comparison of compliant backbone tracking for two examples, *Horizontal*, shown in **(A),** and *Square*, shown in **(B)**. **(A)** Horizontal demand path. **(B)** Square demand path.

In Figure 7, the backbone tracking performance is presented along the length of the backbone for visual confirmation of the continuum robots’ tracking ability. Similarly, only two demand paths are given, namely, the *Horizontal* and *Square* paths. The positions along the backbone that correspond to the locations of the OptiTrack markers are represented by circles. There are multiple positions in time that could be selected for examination, with four selected for reasons of clarity. The four positions aim to cover the maximum spread of the movement to illustrate the comparison between the simulated and experimental data.

This is a visual confirmation of the observations seen above in this study. It can be seen that the continuum robot can track the demand and thus confirm the predictions of the mathematical model. As already seen, the tracking accuracy decreases further from the resting position as the robot moves. In this work, the decision was taken to not use any shape accuracy metric for the entire backbone; instead, tip tracking is considered a good approximation for the overall performance since all other points on the backbone are being traced in a very similar fashion. The information included in backbone tracking clearly shows that the mathematical model can predict the actual backbone with the necessary adjustments, considering physical parameters, such as friction.

## 7 Conclusion

In this work, the development and validation of a feedforward control methodology utilizing a mathematical model based on Cosserat rod theory for a tendon-driven continuum robot is presented. The mathematical model and numerical technique are designed to enable accurate results to be achieved with reduced computation time by employing an implicit time discretization scheme. The ability of the continuum robot to track a prescribed trajectory at the tip position was examined for six different paths, where the motion of the robot was identified using a motion capture system. The results show good trajectory tracking for all paths, with errors less than 9.5% and consistent repeatability between runs.

The proposed approach was not only able to control the tip trajectory of the continuum robot, but it was also able to predict the overall shape of the mechanism as it tracked the demanded path. This work demonstrates that the most common challenges of model-based control for continuum robots, such as the computational complexity of real-time control, can be overcome, and a reliable, precise control methodology can be achieved. With the current capacity demonstrated, endoscopic applications are a promising proposal when utilizing the identified intrinsic parameters of the robot and accounting for a smaller size. This will provide the ability to track arbitrary backbone shapes, providing an efficient and accurate approach for controlling the robot and navigating natural pathways in the body.

## Data Availability

The raw data supporting the conclusion of this article will be made available by the authors, without undue reservation.
